# Multiple Paternity in a Reintroduced Population of the Orinoco Crocodile (*Crocodylus intermedius*) at the El Frío Biological Station, Venezuela

**DOI:** 10.1371/journal.pone.0150245

**Published:** 2016-03-16

**Authors:** Natalia A. Rossi Lafferriere, Rafael Antelo, Fernando Alda, Dick Mårtensson, Frank Hailer, Santiago Castroviejo-Fisher, José Ayarzagüena, Joshua R. Ginsberg, Javier Castroviejo, Ignacio Doadrio, Carles Vilá, George Amato

**Affiliations:** 1 Department of Ecology, Evolution and Environmental Biology, Columbia University, New York, New York, United States of America; 2 Sackler Institute of Comparative Genomics, American Museum of Natural History, New York, New York, United States of America; 3 Fundación Palmarito Casanare, Bogotá, Colombia; 4 Dpto. Biodiversidad y Biología Evolutiva, Museo Nacional de Ciencias Naturales, CSIC, Madrid, Spain; 5 Estación Biológica El Frío, Apure, Venezuela; 6 LSU Museum of Natural Science, Department of Biological Sciences, Louisiana State University, Baton Rouge, Louisiana, United States of America; 7 Department of Evolutionary Biology, Uppsala University, Uppsala, Sweden; 8 School of Biosciences, Cardiff University, Cardiff, CF10 3AX, Wales, United Kingdom; 9 Center for Conservation and Evolutionary Genetics, Smithsonian Conservation Biology Institute, National Zoological Park, Washington, DC, United States of America; 10 Lab. de Sistemática de Vertebrados, Pontifícia Universidade Católica do Rio Grande do Sul (PUCRS), Porto Alegre, Brasil; 11 Cary Institute of Ecosystem Studies, Millbrook, New York, United States of America; 12 Asociación Amigos de Doñana, Seville, Spain; 13 Conservation and Evolutionary Genetics Group, Estación Biológica de Doñana (EBD-CSIC), Seville, Spain; Embrapa, BRAZIL

## Abstract

The success of a reintroduction program is determined by the ability of individuals to reproduce and thrive. Hence, an understanding of the mating system and breeding strategies of reintroduced species can be critical to the success, evaluation and effective management of reintroduction programs. As one of the most threatened crocodile species in the world, the Orinoco crocodile (*Crocodylus intermedius*) has been reduced to only a few wild populations in the Llanos of Venezuela and Colombia. One of these populations was founded by reintroduction at Caño Macanillal and La Ramera lagoon within the El Frío Biological Station, Venezuela. Twenty egg clutches of *C*. *intermedius* were collected at the El Frío Biological Station for incubation in the lab and release of juveniles after one year. Analyzing 17 polymorphic microsatellite loci from 335 hatchlings we found multiple paternity in *C*. *intermedius*, with half of the 20 clutches fathered by two or three males. Sixteen mothers and 14 fathers were inferred by reconstruction of multilocus parental genotypes. Our findings showed skewed paternal contributions to multiple-sired clutches in four of the clutches (40%), leading to an overall unequal contribution of offspring among fathers with six of the 14 inferred males fathering 90% of the total offspring, and three of those six males fathering more than 70% of the total offspring. Our results provide the first evidence of multiple paternity occurring in the Orinoco crocodile and confirm the success of reintroduction efforts of this critically endangered species in the El Frío Biological Station, Venezuela.

## Introduction

Successful reproduction is critical to the recovery of endangered species. Many factors may limit reproduction, but in a small, newly established population knowledge of a species' mating system can be of particular importance to understanding the effective population size and the factors that drive reproductive success more generally [1]. Mating systems and their potential influence in the effective population size are also of critical importance for the design of *ex situ* conservation strategies for the recovery of endangered species [2]. Reintroduction of captive populations has proven challenging with approximately one-third of reintroductions failing [3,4] due to one or a combination of factors including poor habitat quality [5], altered behaviors (e.g. nest site selection, courtship rituals), and depleted genetic diversity [6].

For many species it is difficult, if not impossible, to regularly observe matings. Over the last thirty years, genetic tools have provided an increasingly accurate, and subtle, means to assess mating systems and to examine the result of differential mating success among both males and females [7]. Genetic tools have revealed that, throughout the animal kingdom, females of many species pursue a variety of alternative reproductive strategies exercising a more active role in mate choice [8,9], often mating with more than one male [10,11]. The result of this discovery of widespread polyandry is a better understanding of the frequency of extra pair paternity [12], female cryptic choice [13], and multiple paternity of a clutch or litter of offspring [11].

Multiple paternity of a single clutch, whereby offspring in that clutch are fathered by different males, can be the result of multiple mating within a single breeding season [13] or fertilizations from sperm stored from matings in previous seasons [14]. The evolution of female multiple mating (polyandry), and multiple paternity, can be driven by direct and indirect benefits. Direct benefits include provisioning of resources to the mother and paternal care of offspring [15,16]. Indirect benefits that may accrue include: improved quality of mate resulting in transfer of “good genes” or offspring of higher fitness [17,18], maximization of genetic diversity among the offspring resulting in bet-hedging against variation in the environment in successive generations [19,20], and improved genetic compatibility between mating pairs [21,22]. Mate encounter rates within a reproductive season also have been shown to influence both the occurrence and frequency of multiple paternity [23]. By increasing the number of males that contribute offspring to successive generations, and by changing the frequency of such contributions multiple paternity has also been shown, both theoretically [24] and empirically [25], to increase the effective population size [24], potentially increasing the overall genetic diversity of a population [26].

As a group, crocodilians were long believed to be polygynous and monandrous, with dominant males establishing breeding territories, excluding other adult males, and mating with multiple females [27–29]. This belief was primarily based on observations of crocodilians in captivity, supported with a few, mostly anecdotal, accounts of wild animals [30]. Multi-locus, highly variable, codominant markers such as microsatellites are a powerful genetic tool for parentage analysis [31]. They can be used for reconstruction of full-sibling and half-sibling families and inference of parental genotypes using maximum likelihood frameworks [32]. The use of molecular markers has shown multiple paternity to be an ubiquitous phenomenon [33–39].

Using these methods and data, evidence of multiple paternity has been observed in many crocodilian species, including members of families Crocodylidae [34–37] and Alligatoridae [38,39]. The frequency of multiple-sired nests ranges from 30% to 90% [34–37]. Across studies, multiply sired clutches were fathered by two to four males [34–37]. Paternal contributions to multiply sired clutches were found to be skewed in the American alligator (*Alligator mississippiensis*) [38] and spectacled caiman (*Caiman crocodilus*) [37], with some males significantly contributing more than others to the total reproductive output. Overall, across all species of Crocodylia, these studies reveal high variability both in the prevalence of multiple paternity, measured as the percentage of multiple-sired clutches, and in the relative paternal contribution of males. This variability suggests that the incidence of multiple paternity is likely influenced by a variety of environmental factors as well as species behavior and life history traits.

Captive breeding and reintroduction has been used worldwide as a tool to recover critically endangered species of crocodilians including the Chinese alligator (*Alligator sinensis*) [40], the Cuban crocodile (*Crocodylus rhombifer*) [41], the Philippine crocodile (*Crocodylus mindorensis)* [42], the Siamese crocodile (*Crocodylus siamensis*) [43,44], the gharial (*Gavialis gangeticus*) [45], and the Orinoco crocodile (*C*. *intermedius*) [46]. With the exception of *A*. *sinensis* [40], information about mating systems and occurrence of multiple paternity based on genetic data is lacking. Genetic investigations of reproductive success could improve our understanding of crocodilian reproductive ecology and greatly aid *ex-situ* and *in-situ* conservation strategies, in particular the demographic and population structure of crocodilian reintroduction efforts.

**Fig 1:**
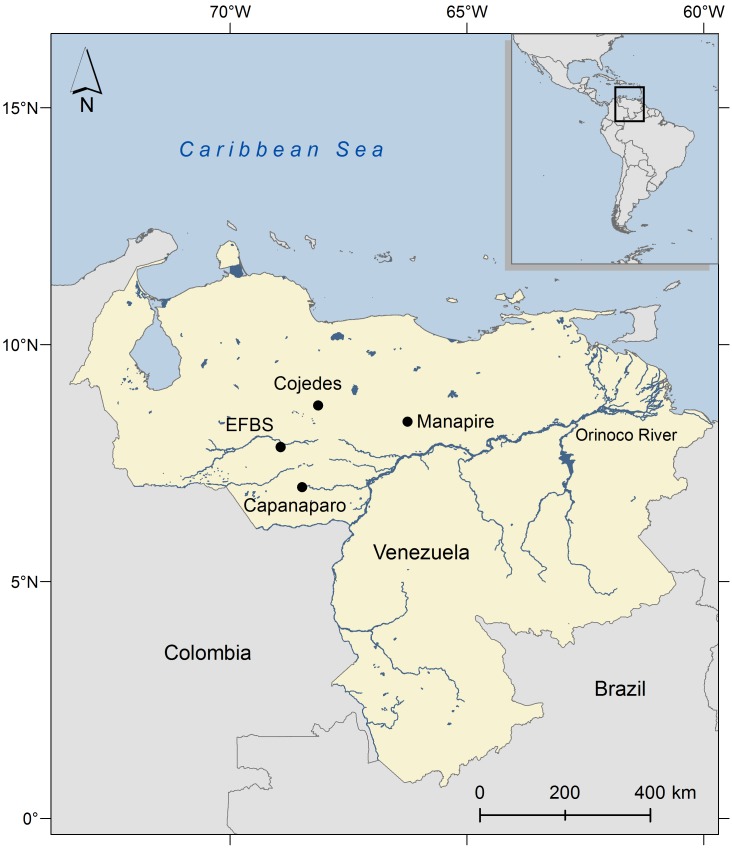
Location of the El Frío Biological Station (EFBS), and three additional localities where last remaining populations of *Crocodylus intermedius* are found in Venezuela.

One of the most threatened crocodile species in the world, the Orinoco crocodile [46,47] once inhabited large areas of the Llanos of Venezuela and Colombia within the Orinoco basin [48,49]. Extensive hunting until the 1960s and persistent collection of eggs for local consumption decimated its populations [50,51], with only few wild populations remaining in both countries [52]. In Venezuela, these include principally natural populations at Capanaparo, Cojedes and Manapire river systems [52], and a reintroduced population at Caño Macanillal and La Ramera lagoon within the El Frío Biological Station (EFBS) (Fig 1), and the adjacent Caño Guaritico Wildlife Refuge [53]. The original population at EFBS was extirpated during the 20^th^century. From 1990–2006 efforts were made to re-establish this population by the reintroduction of more than 2000 crocodiles raised in four captive breeding centers in Venezuela [53]. Post reintroduction, several management interventions were made to improve the survival of the *C*. *intermedius* at EFBS: river sand was supplemented along the shores of rivers and lagoons to facilitate the excavation of nests by females, eggs were collected and artificially incubated to prevent losses from nest predation, with the primary nest predator being the Tegu lizard (*Tupinambis teguixin*), juveniles were retained in captivity in a "head-start" program and released as one year-old juveniles to avoid the highest mortality rates which occur during the first year of life [46,53,54].

The occurrence of male hierarchies in Orinoco crocodiles during the breeding season was observed in previous studies [55,56]. However, both the extent to which males are able to monopolize breeding, and the occurrence of multiple mating, were unknown. Here, we use 17 polymorphic microsatellite loci to determine the occurrence of multiple paternity in 20 clutches of *C*. *intermedius* collected in EFBS, Venezuela. Specifically, we investigated the incidence of multiple paternity over three breeding seasons, used a maximum likelihood approach for sibship and parental inference, and assessed the degree of reproductive skew among inferred fathers.

## Materials and Methods

### Sampling

Twenty clutches of *C*. *intermedius* were collected during three consecutive years (n = 1 in 2004; n = 7 in 2005; and n = 12 in 2006) along artificial nesting sites placed in the wetland shorelines of Caño Macanillal (7° 50' 11.45"N, 68° 55' 43.33"W) and La Ramera lagoon (7° 50' 5.65"N, 68° 55' 38.14"W) in the EFBS, Venezuela (Fig 1). Clutches were transported to the EFBS and incubated in artificially constructed nests as part of a captive breeding and reintroduction program for this species. Upon hatching, skin tissue samples were taken from all hatched crocodiles (n = 335). Samples were removed from the dorsal section of the base of the tail and stored at 8°C in 95% ethanol, following sampling protocols described in [36].

The Oficina Nacional de Diversidad Biológica, Ministerio del Ambiente y de los Recursos Naturales de Venezuela, granted tissue sample collection permits (Permit number 5–0358) and endorsed all other research activities of this project. CITES export permit number 1395/VE9120190 allowed *C*. *intermedius* skin tissue samples to be transported to Uppsala University, Sweden, and Museo Nacional de Ciencias Naturales (CSIC) in Madrid, Spain, to conduct genetic laboratory work.

### Genotyping

Genomic DNA was extracted from preserved scales using the DNeasy blood and tissue kit (Qiagen, Valencia, CA) following manufacturer protocols. A panel of 17 microsatellite markers previously developed for *Crocodylus*, including C391, Cj16, Cj18, Cj101, Cj122, Cj127, CUJ131, Cu5123 [57], Cj109 [58], CpDi13, CpP302, CpP305, CpP314, CpP801, CpP1409, CpP1610, CpP3216 [59], was used to genotype all samples. Microsatellites were amplified in 4 multiplex PCRs (Mix1: CpP302, CpP305, CpP314, CpP1409, CpP3216, CpP1610; Mix2: C391, Cj16, Cj122, CUJ-131; Mix3: Cj18, Cj109, Cu5-123; Mix4: Cj101, Cj127, Cp801, CpDi13) using the Qiagen Multiplex PCR kit (Qiagen, Valencia, CA) for 30 cycles and two different annealing temperatures (57°C for Mix1, Mix2 and Mix4, and 60°C for Mix3). Reactions were prepared in a final volume of 12.5 μL including: 6.25 μL of Qiagen 2X PCR Master Mix, 2.5 μL of 10X primer mix (final concentration: 2 μM each, except for Cj16, Cj127 and CpP801, which were used at 4 μM), 2 μL DNA (ca. 10 ng/μL) and 1.75 μL of RNase-free H_2_O. Fluorescently labeled PCR products were electrophoresed on an ABI 3730 XL DNA analyzer with GS500 (-250) ROX size standard (Applied Biosystems, Foster City, CA) and allele sizes were determined using GeneMapper 3.7 software (Applied Biosystems Foster City, CA). Genetic laboratory work was conducted at Uppsala University, Sweden, and Museo Nacional de Ciencias Naturales, Spain.

The number of alleles, observed and expected heterozygosities, polymorphic information content, exclusion probabilities, allele frequencies, and tests for presence of null alleles were calculated in CERVUS 3.0.7 [60] for a sub-sample of one randomly selected individual per nest (n = 20). CERVUS 3.0.7 [60] uses genetic data from codominant markers to estimate allele frequencies and calculate various summary statistics and exclusion probabilities for each locus, assuming autosomal markers, linkage equilibrium between genetic markers, and diploid species. Conformity to Hardy-Weinberg (HW) expectations for each locus and genotypic linkage disequilibrium (LD) between pairs of loci were tested in GENEPOP 4.3 [61]. Significance levels (p = 0.05) for departure from HW and LD were corrected for multiple comparisons with a sequential Bonferroni correction [62].

Contamination and primer-site mutations may result in the incorrect assignment of microsatellite genotypes (genotyping errors), potentially biasing the results of parentage analysis [63]. Additionally, DNA degradation, low DNA concentration, and primer-site mutations can potentially cause non-amplified alleles (null alleles), which have proven to introduce errors in parentage analysis leading to high frequencies of erroneous parentage exclusions [64]. Potential genotyping inconsistencies including the presence of null alleles, large allele dropout, scoring of stutter peaks and typographic errors, were assessed in Micro-checker [65] and specific allele dropout rates were estimated in Microdrop 1.10 [66].

### Paternity analysis

Sibship and parental inference analyses were conducted using a maximum likelihood approach implemented in COLONY 2.0 [32]. COLONY assigns individuals into full-sib and half-sib families using multi-locus genetic marker data with or without the imputing of parental information [32], and allowing for the incorporation of various types of genotyping errors [67]. By clustering offspring into full- and half-sib families COLONY is able to infer parental genotypes, when unknown, and calculate paternity assignment likelihoods, assuming Mendelian segregation and no maximum limit on the numbers of contributing parents. Ten replicate runs of “long” length and “high” likelihood precision were carried out in COLONY using the same dataset assuming an error rate of 0.04 for allelic dropout and 0.05 for genotyping error (based on the highest error rate per locus obtained in Microdrop 1.10 [68] (S1 Table) and suggested genotyping error rates by [67]). Polygamy was assumed for males and females and a full likelihood method was implemented. Offspring genotypes and known maternal sibship for each clutch were inputted into COLONY.

We used GERUD 2.0 [69] as alternative analysis package for the detection of multiple paternity. GERUD uses an exhaustive algorithm to determine the minimum number of fathers that can explain a full or half-sib progeny array when one or neither parent is known [69]. Because GERUD can only incorporate up to 10 loci, we used the 10 loci with highest exclusion probabilities (Table 1).

**Table 1:** Characterization of 17 microsatellite loci in *Crocodylus intermedius* at the El Frío Biological Station, Venezuela.

Locus	n	Allele size range (bp)	No. Alleles	H_o_	H_E_	PIC	PE	PrDM
CpP1409	20	231–238	4	0.464	0.371	0.310	0.073	
CpP1610	20	274–278	2	0.143	0.135	0.124	0.012	
CpP302	20	168–204	10	0.821	0.862	0.829	0.559	
CpP305	20	160–182	5	0.536	0.531	0.484	0.109	
CpP314	20	236–250	6	0.500	0.592	0.516	0.184	
CpP3216	20	121–125	2	0.464	0.503	0.372	0.120	
C391	20	164–196	11	0.815	0.807	0.762	0.362	
CUJ131	20	202–210	4	0.536	0.551	0.474	0.154	
Cj122	20	392–406	4	0.667	0.713	0.645	0.250	
Cj16	20	160–194	7	0.679	0.722	0.660	0.264	
Cj109	20	380–408	11	0.750	0.774	0.733	0.317	
Cj18	20	225–235	5	0.571	0.573	0.481	0.167	
Cu5123	20	200–218	4	0.286	0.249	0.215	0.030	
Cj101	20	378–388	4	0.464	0.551	0.442	0.119	
Cj127	20	351–357	3	0.179	0.166	0.149	0.024	
CpDi13	20	342–348	3	0.500	0.499	0.370	0.085	
CpP801	20	164–184	5	0.538	0.651	0.571	0.270	
**Overall**	** **	** **	** **	**0.524**	**0.544**	**0.479**	**0.976**	**0.999/0.876**

H_0_: observed heterozygosity; H_E_: expected heterozigosity; PIC: polymorphic information content; PE: probability of excluding a potential parent when neither parent is known; PrDM: probability of detecting multiple paternity given 17 progeny sampled per clutch and two fathers contributing 50:50 / 90:10.

Probabilities of detecting multiple paternity were estimated using the PrDM software [70] (http://publish.uwo.ca/~bneff/software.html) for scenarios of equal (50:50) or skewed (90:10) paternal contribution by two males, averaged across 10 replicate runs, and using an average of 17 hatchlings per clutch sampled in this study. The degree of reproductive skew (B) was assessed for clutches exhibiting multiple paternity by calculating the binomial skew index B [71–73] in SKEW CALCULATOR 2003 (https://www.eeb.ucla.edu/Faculty/Nonacs/PI.html). Significant levels of B were calculated by simulation with 100,000 permutations. Significant positive values of B indicate a skewed paternal contribution to a given clutch, significant negative values of B indicate an excessively equal paternal contribution, and non significant values do not show differences from a random paternal contribution [71]. A Pearson's product moment correlation test between number of alleles per locus and exclusion probabilities, clutch size and number of fathers, and clutch size and probability of detecting multiple paternity (PrDM) was conducted using the package stats in R version 3.1.2 [74].

## Results

### Characterization of microsatellite markers

We identified a total of 90 alleles averaging 5.3 alleles per locus among a subsample of 20 Orinoco crocodile individuals, taking a random individual from each clutch. Observed and expected heterozygosities ranged from 0.143 and 0.135 for CpP1610, respectively, to 0.821 and 0.862 for CpP302, exhibiting overall values of 0.524 and 0.544, respectively. Our analysis did not detect significant (p<0.05) deviations from Hardy-Weinberg expectations and linkage equilibrium at any locus (S1 Table), or evidence of null alleles.

The discriminating power of the 17 markers combined was high (Table 1), with a probability of excluding a potential parent when neither parent is known (PE) of 0.976 and probabilities of detecting multiple paternity (PrDM) given 17 offspring sampled per clutch and two fathers contributing 50:50 and 90:10 of 0.999 and 0.876, respectively. However, exclusion probabilities (PE) of individual markers were mostly low, ranging from 0.012 to 0.559, and were highly correlated with the number of alleles per locus (r = 0.831).

On a clutch-by-clutch basis, probabilities of detecting multiple paternity (PrDM) remained relatively high (p>0.830) for all clutch sizes under the equal paternal contribution scenario, and ranged from 0.587 to 0.852 under a skewed paternal contribution (90:10) (Table 2).

**Table 2:** Paternity analyses for 20 clutches of *Crocodylus intermedius* from the El Frío Biological Station, Venezuela.

				GERUD	COLONY								
Clutch ID	Year	N	PrDM	Number of inferred fathers	Inferred mother ID	Number of inferred fathers	Paternal sibship	Inferred father ID	N full siblings	Prob (Inc.)	Prob (Exc.)	*B* value*	p
C1	2004	3	0.747/0.546	1	M1	1	1A	F1	3	1.000	1.000		
C2	2005	16	0.952/0.788	2	M2	2	2A	F2	15	1.000	1.000	**0.3516**	**0.0005**
							2B	F3	1	1.000	1.000		
C3	2005	32	0.925/0.744	1	M3	1	3A	F4	32	1.000	1.000		
C4	2005	19	0.983/0.801	2	M4	2	4A	F5	13	1.000	1.000	0.0416	0.1679
							4B	F1	6	0.986	0.986		
C5	2005	19	0.972/0.776	1	M5	1	5A	F2	19	0.604	0.604		
C6	2005	8	0.944/0.734	3	M1	2	6A	F1	7	1.000	1.000	0.2188	0.0684
							6B	F6	1	1.000	1.000		
C7	2005	8	0.971/0.768	2	M6	2	7A	F2	7	0.999	0.999	0.2188	0.0708
							7B	F3	1	1.000	1.000		
C8	2005	42	0.996/0.852	1	M7	1	8A	F2	42	1.000	1.000		
C9	2006	14	0.972/0.971	3	M8	3	9A	F7	9	0.949	0.904	** **	** **
							9B	F8	4	0.811	0.797		
							9C	F2	1	1.000	1.000		
C10	2006	24	0.957/0.743	1	M9	1	10A	F2	24	1.000	1.000		
C11	2006	8	0.980/0.798	1	M5	1	11A	F2	8	1.000	1.000		
C12	2006	22	0.931/0.706	2	M10	2	12A	F9	21	1.000	1.000	**0.3905**	**0.0000**
							12B	F10	1	1.000	1.000		
C13	2006	23	0.940/0.718	1	M11	1	13A	F7	23	1.000	1.000		
C14	2006	3	0.929/0.746	2	M6	2	14A	F2	2	0.999	0.999	−0.1111	1.0000
							14B	F6	1	1.000	1.000		
C15	2006	13	0.983/0.807	3	M7	3	15A	F2	11	1.000	1.000	**0.3432**	**0.0007**
							15B	F3	1	1.000	1.000		
							15C	F11	1	1.000	1.000		
C16	2006	19	0.897/0.653	1	M12	1	16A	F12	19	0.998	0.998		
C17	2006	16	0.954/0.745	1	M13	1	17A	F7	16	1.000	1.000		
C18	2006	23	0.902/0.677	3	M14	3	18A	F13	21	1.000	1.000	**0.4751**	**0.0000**
							18B	F7	1	1.000	1.000		
							18C	F14	1	1.000	1.000		
C19	2006	16	0.950/0.738	1	M15	1	19A	F7	16	1.000	1.000		
C20	2006	7	0.830/0.587	2	M16	2	20A	F10	4	0.706	0.706	−0.0612	1.0000
							20B	F11	3	0.549	0.549		

N: number of offspring used in paternity analysis; PrDM: probability of detecting multiple paternity given two fathers contributing 50:50/90:10, averaged over 10 replicate simulations; Prob (Inc.): probability that all individuals of a given full-sib paternal family are full-sibs; Prob (Exc.): probability that no other individuals are full-sibs within this paternal family; B value: binomial skew index; p: significance level of B value.

*Significant values of B are represented in bold.

### Paternity analysis

We obtained complete multilocus genotypes for all of the 17 loci and for more than 95% of the total individuals (335) sampled within a total of 20 clutches. Genotype data can be accessed at the Dryad Digital Repository under the DOI: 10.5061/dryad.n2114. Multiple paternity and parental reconstruction results were consistent over 10 COLONY replicate runs, assuming error rates of 0.04 for allelic dropout and 0.05 for genotyping error, and using different random seed numbers for each run. Our analyses revealed evidence of multiple paternity, with 10 (50%) out of 20 clutches fathered by two or three males (Table 2). We found multiple paternity in two of the three breeding seasons analyzed (2005 and 2006), with four out of seven clutches (57%) in 2005, and six out of 12 clutches (50%) in 2006 being fathered by two or three males. The only nest from 2004 was single-sired. GERUD and COLONY inferred multiple fathers for the same 10 clutches. The number of fathers remained the same using both programs for all but one clutch (C6), for which GERUD determined a minimum of three fathers whereas COLONY determined a minimum of two fathers. Due to the different number of markers used (a maximum of 10 loci for GERUD and all 17 for COLONY), the estimate offered by COLONY is considered more reliable.

For 90% of clutches, configurations of full-sib families inferred by COLONY exhibited high probabilities of both including all full-sib individuals in a given paternal family (Prob Inc.), and excluding all non full-sibs from each paternal family (Prob Exc.). Clutches 5 and 20 showed the lowest probabilities for full-sib paternal family configurations. Potential causes of these low values may be associated with mislabeling of hatchlings or mutations at one or several of the microsatellite loci that might have generated mismatches among sib hatchling genotypes.

Of the 10 multiple-sired clutches, four (40%) were significantly skewed from equal paternal contributions (Table 2). Four additional clutches (C6, C7, C14, C20) failed to show significant skew between fathers, which may be explained by small clutch size (n<10) hindering the degree of reproductive skew. We did not find significant correlations between clutch size and number of fathers (t = -1.639, df = 21, p = 0.116), and clutch size and probability of multiple paternity (PrDM) (t = -1.328, df = 21, p = 0.199).

COLONY reconstructed 16 maternal and 14 paternal multilocus genotypes (S2 Table). Four of the 16 inferred mothers laid eggs on consecutive nesting seasons (M1, M5, M6, and M7), with two of these (M1 and M6) returning to the same geo-referenced nesting site (data not shown). Full siblings were observed in different nests when females mated with the same male in successive years. Some of these females laying eggs over consecutive seasons mated exclusively with the same male (M5), but the majority mated with more than one male in either one (M1, M7) or two (M6) consecutive breeding seasons.

**Fig 2:**
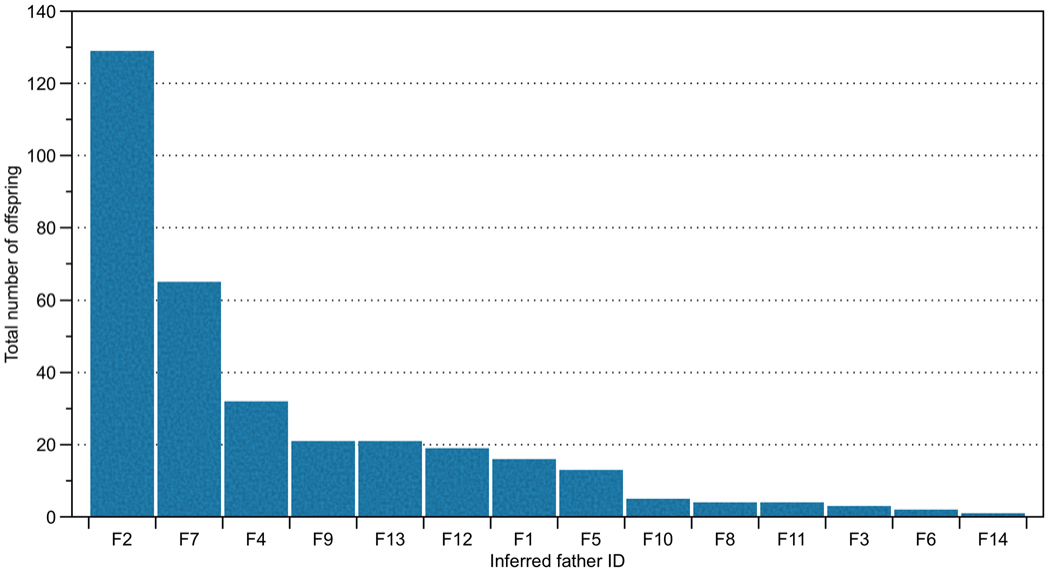
Paternal contributions from 14 fathers inferred by COLONY for 335 hatchlings of *Crocodylus intermedius*.

Shared paternity across clutches was inferred by COLONY, with highly skewed contributions among identified males (Fig 2). Six of the 14 inferred males (F2, F4, F7, F9, F12 and F13) fathered 90% of the total offspring, and three of those six males (F2, F4 and F7) fathered more than 70% of the total offspring.

## Discussion

Our results report for the first time a polygamous mating system for *C*. *intermedius*, with females mating with more than one male in 10 out of 20 clutches studied. *Crocodylus intermedius* at the EFBS exhibited both single-fathered and multiple-fathered clutches, indicating that females can employ different reproductive strategies. Results of this study accord with other studies of multiple paternity in crocodilians including 30% multiple-sired clutches in *A*. *sinensis* [39], 32% in *A*. *mississippiensis* [38], 50% in *Crocodylus moreletii* [33], 50% in *Caiman latirostris* [34], 69% in *Crocodylus porosus* [36], 90% in *Melanosuchus niger* [35], and 95% in *C*. *crocodilus* [37]. In these studies, two to four males fathered multiply sired clutches.

Observed differences in the incidence of multiple paternity within and among crocodilian species could be influenced by one or a combination of factors, including: density of breeding individuals, mate encounter rates, sex ratio of sexually mature individuals, differences in mating behavior and reproductive strategies (e.g. ability of males to monopolize matings, male territory defense during the breeding season, male harassment, female choice, timing of copulation), and habitat type and spatial configuration of the environment (e.g. mangrove swamps, internal rivers and lagoons, small streams, ponds). Relatively small sample sizes, and a dearth of comparative data, make it difficult to identify which of these factors come in to play between and among these studies.

Multiple paternity in *C*. *intermedius* could have resulted from multiple mating within a single breeding season and/or fertilizations from sperm stored in females’ reproductive tract within one or more breeding seasons. Sperm storage has proven an effective strategy to ensure fertilization and lengthen the breeding season in taxa where rates of encounter between the sexes are low, multiple clutches are laid within a single reproductive season, and/or where high asynchrony in gonadal cycles between males and females occurs [38,75,76]. Although sperm storage has not been reported in *C*. *intermedius*, sperm was found in the oviducts of female *A*. *mississippiensis* [77] and possibly occurred in a captive female of *Paleosuchus palpebrosus* which laid fertile eggs a long time after being separated from a male [78]. The absence of sperm from the oviduct of female American alligators during non-reproductive periods, however, suggested that alligator sperm may be retained within a reproductive season but is unlikely to be retained from one year to the next [77]. Female crocodilians generally congregate in breeding areas with groups of males during the reproductive season [79–81], lay one clutch per reproductive season on an annual or bi-annual basis [30], and some species showed a degree of synchrony in gonadal cycles between males and females (*A*. *mississippiensis* [82] and *C*. *niloticus* [83]). Given low population numbers, female sperm storage in *C*. *intermedius* (if it does occur) would most likely be associated with low frequency of mate encounter and low mate availability. Female cryptic choice (female selection for a particular male’s sperm [84]) and the assurance of paternal diversity within clutches [85] could also potentially result in sperm storage in this species.

Our results, and observations from previous studies documenting male hierarchies in Orinoco crocodiles during the breeding season [55,56], suggest unequal distribution of matings. In this study shared paternity across clutches revealed highly skewed contributions among identified males. Similarly, paternal contributions of multiply sired clutches were skewed in *A*. *mississippiensis* [86] and *C*. *crocodilus* [37]. Skewed paternal contributions to clutches may arise by one or a combination of different mechanisms, such as: female preference for a “dominant” male, higher success for copulations occurring closest to female ovulation, sperm storage and female cryptic choice, and competition among male’s sperm in female’s reproductive tract (sperm competition [87]).

Four of the 16 identified mothers returned to lay eggs in two consecutive years, with two of them laying eggs in the same nesting site on the second year. In this recently reintroduced population, because of a lack of appropriate habitat, nesting sites have been artificially supplemented by providing river sand along the shores of rivers and lagoons to facilitate the excavation of nests by females. Despite the artificial construction of nest sites, two females showed site fidelity, a phenomenon not uncommon to crocodilians [44]. Of the four returning females, two exhibited a switch from a single-sired clutch in one breeding season to a multiple-sired clutch in the subsequent breeding season, whereas the other two maintained either single-sired or multiple-sired clutches in both breeding seasons (Table 2). Hence, in this small sample, mating strategy, nesting site, and site fidelity, do not appear correlated, although further research is required to parse out the reasons for site fidelity.

A recent study of *C*. *acutus* [88] reporting nests with full siblings in successive mating seasons suggests the occurrence of mate fidelity in this species. In this study, we found that all four females that laid clutches on two consecutive seasons mated with at least one of the same males. Of these, only one mated exclusively with a single male, whereas the other three had multiple mates in either one or two breeding seasons. These findings could be a consequence of female choice and/or male dominance within this population.

Inference of parental genotypes from offspring multilocus marker data and derived interpretations needs to be carefully assessed. Maximum likelihood approaches for sibship and parental inference implemented by COLONY 2.0 [32] have been shown to overestimate parent numbers in both empirical [89] and simulated marker datasets [90] with low to intermediate levels of polymorphism. Nonetheless, parentage studies and family reconstructions of simulated datasets identified number and diversity of loci as main factors determining accuracy of results, with COLONY outperforming most available methods of parentage analysis [91]. In this study, the resolving power of the combined marker set was high. In addition, convergence of multiple runs in COLONY and high values of full-sib family exclusion and inclusion probabilities for the majority of the clutches suggested that our marker set could be sufficient for accurate parental reconstruction. The alternative method used to assess multiple paternity (GERUD) accorded with COLONY and confirmed the robustness of our analysis.

Information on the mating system and breeding strategies of reintroduced populations is critical to their success, evaluation and effective management [1]. The occurrence of multiple paternity may accelerate the recovery and resilience of reintroduced populations of *C*. *intermedius* by increasing genetic diversity among siblings, potentially increasing the effective population size [24], and overall genetic diversity of the population [26]. However, the dominance of certain males could potentially have the opposite effect by reducing the number of contributing males and increasing the variance in their contribution to the gene pool of next generations. In this study, we report heterozygosity values similar to those of other crocodilian populations in the wild [92–94]. The latter suggests that the genetic makeup and/or rapid population growth after individuals were reintroduced may have quickly compensated any potential founder effect, despite biases in the contribution of the different males.

Before the first reintroductions of *C*. *intermediu*s, the species had been depleted from the El Frío Biological Station and surrounding areas [53] that fall within the historical range of the species [48,49]. After first reintroduction efforts took place in 1990, a combination of management interventions resulted in a successful population recovery with increasing nesting numbers adding up to at least 93 nests between 1996 and 2007 [53]. Nesting at the EFBS lays within nesting numbers previously reported at other sites: in the Capanaparo river system there were 11 nests in 1991 [81], 14 nests in 2001, 20 nests in 2002 [47], and 25 nests in 2013 [95]; during the 2009 nesting season 13 nests were laid in the Cojedes [52] and four nests in the Manapire river systems [47]. In the context of the few remaining natural populations of *C*. *intermedius* in Venezuela, the EFBS reproductive output makes it a very important population for the recovery of the species.

Successful reproduction is a necessary first step in the recovery of the Orinoco crocodile. However, other conservation actions will be critical to the continued and sustained growth of this and other populations. Conservation actions include mitigating the threat of poaching and habitat protection [50]. Continued involvement and support from the governmental nature conservation authorities is essential for the long-term sustainability of these initiatives. Political changes in Venezuela led to a reduction in conservation and research activities at the El Frío Biological Station in 2009. Since then, the fate of the reintroduced Orinoco crocodile population, one of the last populations of C. *intermedius*, is little known. The limited information published in the popular press in recent years suggests that this population, and the species more generally, continues to be in need of urgent conservation actions [96].

Future research should aim to better elucidate the factors that influence multiple paternity in crocodilian species, looking at environmental (e.g. latitude, temperature, habitat type and configuration), demographic and species-specific traits. Research on specific mechanisms involved in producing multiple-fathered clutches, potential long-term adaptive advantages of multiple paternity for crocodilians, and impacts of multiple paternity on the overall genetic diversity of populations will further our knowledge on crocodilian mating systems and can aid conservation strategies for endangered crocodilian species.

## Supporting Information

S1 Table Additional information on 17 microsatellite loci used in *Crocodylus intermedius* paternity analysis. (DOC)

S2 Table Parental genotypes reconstructed for 20 *Crocodylus intermedius* clutches across 17 polymorphic microsatellite loci. (DOC)
