# Supplementary material for: Multiple Paternity in a Reintroduced Population of the Orinoco Crocodile (Crocodylus intermedius) at the El Frío Biological Station, Venezuela
Source: PLoS One. 2016 Mar 16;11(3):e0150245. doi: 10.1371/journal.pone.0150245 (PMC4794145; doi:10.1371/journal.pone.0150245)
Supplement: S1 Table — (DOC) [file pone.0150245.s001.doc]

**Supplementary Table S1. Additional information on 17 microsatellite loci used in *C. intermedius* paternity analysis.**

| **Locus** | **Primer sequences (5’-3’)** | **Multiplex Mix** | **Ta (˚C)** | **n** | **HW (p-value)** | **SE** | **Allele dropout rate** |
| --- | --- | --- | --- | --- | --- | --- | --- |
| CpP1409 | F: GTTTATGCCCTACTGGTTATCTATC | 1 | 57˚C | 25 | 0.44370 | 0.00000 | 0.00000 |
|  | R: CAGTCGGGCGTCATCAGGGAAGGGGATTTAATAAT |  |  |  |  |  |  |
| CpP1610 | F: CAGTCGGGCGTCATCATAGAGGGATTTTGACTGT | 1 | 57˚C | 25 | 1.00000 | 0.00000 | 0.00004 |
|  | R: GTTTGATTATTTTGTCTGGGTTCTT |  |  |  |  |  |  |
| CpP302 | F: GTTTGGAACCCAAGAACTTACAAC | 1 | 57˚C | 25 | 0.26360 | 0.00160 | 0.00000 |
|  | R: CAGTCGGGCGTCATCATTGGGTTTAGTCAGCACATA |  |  |  |  |  |  |
| CpP305 | F: GTTTGTAGCTGGAACCTGATAGTG | 1 | 57˚C | 25 | 0.27330 | 0.00000 | 0.00000 |
|  | R: CAGTCGGGCGTCATCAGGTTAACACGTGGTAACTACA |  |  |  |  |  |  |
| CpP314 | F: GTTTGAAATGCCACTAATACACACA | 1 | 57˚C | 25 | 0.11840 | 0.00000 | 0.00323 |
|  | R: CAGTCGGGCGTCATCACCAATTCTTCAGGTCCTTAT |  |  |  |  |  |  |
| CpP3216 | F: CAGTCGGGCGTCATCAGATTAATTCATTGGCTCTC | 1 | 57˚C | 25 | 0.71670 | 0.00000 | 0.00016 |
|  | R: GTTTATGCCTTTGCCTTTAG |  |  |  |  |  |  |
| C391 | F: ATGAGTCAGGTGGCAGGTTC | 2 | 57˚C | 25 | 0.57390 | 0.00700 | 0.00000 |
|  | R: CATAAATACACTTTTGAGCAGCAG |  |  |  |  |  |  |
| CUJ131 | F: GTCCCTTCCAGCCCAAATG | 2 | 57˚C | 25 | 0.70140 | 0.00000 | 0.00164 |
|  | R: CGTCTGGCCAGAAAACCTGT |  |  |  |  |  |  |
| Cj122 | F: GTTTCATGCTGACTGTTTCTAATCACC | 2 | 57˚C | 25 | 0.58420 | 0.00000 | 0.04368 |
|  | R: GGAACTACAATTGGTCAACCTCAC |  |  |  |  |  |  |
| Cj16 | F: CATGCAGATTGTTATTCCTGATG | 2 | 57˚C | 25 | 0.92570 | 0.00040 | 0.00729 |
|  | R: TGTCATGGTGTCAATTAAACTC |  |  |  |  |  |  |
| Cj109 | F: GTATTGTCAACCCCACCGTGTC | 3 | 60˚C | 25 | 0.21000 | 0.00270 | 0.00000 |
|  | R: GTTTCCCCTCCACAGATTTACTTGC |  |  |  |  |  |  |
| Cj18 | F: ATCCAAATCCCATGAACCTGAGAG | 3 | 60˚C | 25 | 0.72870 | 0.00100 | 0.00000 |
|  | R: CCGAGTGCTTACAAGAGGCTGG |  |  |  |  |  |  |
| Cu5123 | F: GGGAAGATGACTGGAAT | 3 | 60˚C | 25 | 1.00000 | 0.00000 | 0.00000 |
|  | R: AAGTGATTAACTAAGCGAGAC |  |  |  |  |  |  |
| Cj101 | F: ACAGGAGGAATGTCGCATAATTG | 4 | 57˚C | 25 | 0.58650 | 0.00000 | 0.03461 |
|  | R: GTTTATACCGTGCCATCCAAGTTAG |  |  |  |  |  |  |
| Cj127 | F: CCCATAGTTTCCTGTTACCTG | 4 | 57˚C | 25 | 1.00000 | 0.00000 | 0.00000 |
|  | R: GTTTCCCTCTCTGACTTCAGTGTTG |  |  |  |  |  |  |
| CpDi13 | F: GTTTGTGTCAGCCTATACATGTT | 4 | 57˚C | 25 | 1.00000 | 0.00000 | 0.00000 |
|  | R: CAGTCGGGCGTCATCAGTCTCAGAGTATGCCTAGAA |  |  |  |  |  |  |
| CpP801 | F: CAGTCGGGCGTCATCATTGGCATTAGATTGGTAGAC | 4 | 57˚C | 25 | 0.07370 | 0.00060 | 0.00041 |
|  | R: GTTTCTATGCCAAAGCTACAAC |  |  |  |  |  |  |

Ta, annealing temperature; HW, p-value estimates for Hardy-Weinberg equilibrium test; SE, standard error for p-value estimates of Hardy-Weinberg equilibrium test.
