# Supplementary material for: Multiple Paternity in a Reintroduced Population of the Orinoco Crocodile (Crocodylus intermedius) at the El Frío Biological Station, Venezuela
Source: PLoS One. 2016 Mar 16;11(3):e0150245. doi: 10.1371/journal.pone.0150245 (PMC4794145; doi:10.1371/journal.pone.0150245)
Supplement: S2 Table — (DOC) [file pone.0150245.s002.doc]

**Supplementary Table S2. Parental genotypes reconstructed for 20 *C. intermedius* clutches across 17 polymorphic microsatellite loci.**

| **Father (F) or Mother ID (M)** | **CpP1419** | **CpP1610** | **CpP302** | **CpP305** | **CpP314** | **CpP3216** | **C391** | **CUJ131** | **Cj122** | **Cj16** | **Cj109** | **Cj18** | **Cu5123** | **Cj101** | **Cj127** | **CpDi13** | **CpP801** | **p** |
| --- | --- | --- | --- | --- | --- | --- | --- | --- | --- | --- | --- | --- | --- | --- | --- | --- | --- | --- |
| F1 | 231/235 | 278/278 | 189/200 | 178/180 | 238/246 | 125/125 | 170/182 | 202/202 | 392/405 | 170/187 | 403/405 | 230/230 | 215/215 | 378/382 | 353/353 | 345/345 | 172/172 | **0.917** |
| F2 | 235/235 | 278/278 | 183/189 | 176/180 | 246/246 | 125/125 | 180/192 | 208/208 | 398/402 | 187/191 | 386/388 | 227/230 | 215/215 | 387/387 | 353/353 | 347/347 | 168/168 | **1.000** |
| F3 | 235/235 | 278/278 | 189/202 | 180/180 | 242/246 | 121/125 | 182/182 | 208/208 | 405/405 | 170/187 | 388/398 | 227/232 | 211/215 | 382/387 | 353/353 | 345/347 | 168/172 | **0.630** |
| F4 | 231/235 | 278/278 | 185/202 | 180/180 | 246/246 | 121/125 | 170/182 | 202/208 | 392/405 | 160/187 | 386/388 | 227/230 | 215/215 | 382/382 | 353/353 | 345/345 | 168/172 | **0.703** |
| F5 | 235/235 | 278/278 | 189/189 | 180/180 | 246/246 | 121/125 | 182/182 | 208/208 | 392/392 | 187/187 | 388/386 | 227/227 | 215/215 | 382/382 | 353/353 | 345/347 | 164/164 | **0.926** |
| F6 | 235/235 | 278/278 | 168/202 | 160/180 | 242/246 | 125/125 | 170/182 | 208/208 | 405/405 | 160/187 | 388/390 | 227/227 | 215/215 | 382/387 | 353/353 | 345/345 | 168/172 | **0.632** |
| F7 | 231/235 | 278/278 | 189/200 | 178/180 | 238/246 | 125/125 | 170/182 | 202/202 | 405/405 | 170/187 | 403/405 | 230/230 | 215/215 | 372/382 | 353/353 | 345/345 | 172/172 | **1.000** |
| F8 | 235/235 | 278/278 | 193/202 | 180/178 | 238/246 | 125/125 | 176/182 | 208/208 | 392/405 | 187/187 | 386/388 | 227/227 | 211/215 | 382/382 | 353/353 | 345/345 | 168/168 | **0.950** |
| F9 | 231/235 | 278/278 | 189/200 | 178/180 | 238/246 | 121/125 | 180/182 | 202/208 | 392/405 | 170/187 | 403/405 | 225/230 | 215/215 | 382/387 | 353/353 | 345/347 | 168/172 | **1.000** |
| F10 | 235/235 | 278/278 | 185/202 | 180/180 | 238/242 | 121/125 | 188/196 | 202/208 | 402/402 | 187/187 | 386/388 | 227/227 | 215/215 | 387/387 | 353/353 | 345/347 | 164/168 | **0.782** |
| F11 | 235/235 | 278/278 | 183/189 | 176/180 | 246/246 | 125125 | 183/189 | 208/208 | 398/402 | 187/191 | 386/388 | 227/230 | 215/215 | 387/387 | 353/353 | 347/347 | 168/168 | **0.848** |
| F12 | 231/235 | 278/278 | 202/202 | 160/178 | 242/242 | 121/125 | 170/184 | 210/210 | 392/392 | 160/187 | 398/398 | 227/230 | 215/215 | 382/382 | 353/353 | 345/345 | 168/172 | **0.631** |
| F13 | 235/235 | 278/278 | 185/202 | 180/180 | 246/246 | 121/125 | 180/182 | 208/208 | 392/405 | 170/187 | 396/388 | 227/227 | 215/215 | 382/382 | 353/356 | 345/347 | 168/172 | **0.903** |
| F14 | 231/235 | 278/278 | 202/202 | 160/180 | 242/246 | 125/125 | 182/182 | 208/208 | 402/402 | 187/187 | 386/398 | 227/232 | 211/215 | 382/382 | 353/356 | 345/347 | 168/168 | **0.950** |
| M1 | 235/235 | 278/278 | 168/189 | 160/180 | 238/246 | 121/121 | 180/182 | 202/208 | 392/405 | 160/170 | 388/388 | 227/227 | 211/215 | 382/387 | 353/353 | 345/345 | 168/172 | **0.908** |
| M2 | 231/235 | 278/278 | 183/185 | 178/180 | 242/246 | 121/125 | 170/182 | 202/208 | 398/398 | 187/189 | 380/396 | 227/230 | 200/215 | 382/382 | 353/353 | 345/345 | 168/176 | **0.966** |
| M3 | 231/235 | 278/278 | 189/200 | 178/180 | 238/246 | 125/125 | 180/182 | 202/208 | 392/405 | 170/187 | 403/405 | 225/230 | 215/215 | 382/387 | 353/353 | 345/347 | 168/172 | **0.703** |
| M4 | 235/235 | 278/278 | 168/202 | 180/182 | 238/246 | 121/121 | 182/182 | 208/208 | 392/398 | 160/187 | 388/388 | 227/227 | 215/215 | 382/387 | 353/353 | 345/345 | 168/172 | **0.930** |
| M5 | 231/235 | 274/278 | 202/204 | 178/180 | 238/246 | 121/121 | 180/182 | 208/208 | 392/405 | 170/187 | 380/388 | 227/227 | 215/215 | 382/387 | 353/353 | 345/345 | 168/172 | **0.982** |
| M6 | 235/235 | 278/278 | 187/189 | 180/182 | 238/246 | 125/125 | 166/166 | 202/208 | 392/392 | 170/187 | 386/386 | 227/227 | 215/215 | 382/387 | 353/356 | 345/345 | 168/172 | **0.972** |
| M7 | 231/231 | 278/278 | 189/202 | 178/180 | 238/250 | 125/125 | 166/182 | 208/208 | 398/405 | 160/187 | 388/388 | 230/232 | 211/215 | 382/387 | 353/353 | 345/347 | 168/172 | **0.999** |
| M8 | 235/235 | 278/278 | 183/185 | 180/180 | 242/246 | 121/125 | 166/170 | 202/208 | 392/392 | 160/160 | 386/388 | 227/230 | 215/215 | 382/382 | 353/356 | 345/347 | 168/172 | **0.935** |
| M9 | 231/235 | 278/278 | 189/202 | 160/160 | 246/246 | 121/125 | 170/182 | 208/210 | 392/402 | 187/187 | 384/388 | 230/230 | 211/215 | 382/382 | 353/356 | 345/347 | 164/172 | **0.999** |
| M10 | 235/235 | 278/278 | 168/200 | 180/180 | 242/242 | 121/125 | 166/180 | 208/208 | 392/392 | 170/170 | 386/388 | 230/232 | 215/215 | 382/382 | 353/353 | 347/347 | 168/172 | **0.761** |
| M11 | 231/235 | 278/278 | 189/191 | 160/180 | 238/246 | 121/125 | 176/182 | 202/202 | 405/405 | 160/160 | 388/407 | 227/230 | 211/215 | 382/382 | 353/353 | 345/345 | 164/172 | **0.999** |
| M12 | 235/235 | 278/278 | 187/189 | 180/182 | 238/246 | 125/125 | 166/166 | 202/208 | 392/392 | 170/187 | 386/386 | 227/227 | 211/215 | 382/387 | 353/356 | 345/345 | 168/172 | **0.631** |
| M13 | 231/235 | 278/278 | 185/193 | 160/178 | 246/246 | 121/125 | 176/182 | 208/208 | 392/402 | 187/187 | 386/405 | 227/227 | 215/215 | 382/382 | 353/353 | 345/347 | 164/176 | **0.991** |
| M14 | 235/235 | 278/278 | 185/202 | 180/180 | 246/246 | 121/121 | 166/176 | 208/208 | 392/405 | 160/187 | 386/386 | 227/227 | 215/215 | 382/387 | 353/353 | 347/347 | 164/168 | **0.745** |
| M15 | 235/235 | 274/278 | 189/202 | 180/180 | 246/246 | 121/125 | 182/192 | 208/210 | 392/392 | 187/187 | 398/405 | 230/232 | 215/215 | 382/387 | 353/356 | 347/347 | 168/176 | **0.999** |
| M16 | 235/235 | 278/278 | 193/202 | 180/180 | 238/246 | 121/125 | 170/182 | 202/208 | 392/392 | 187/187 | 388/405 | 230/230 | 211/215 | 382/382 | 353/353 | 345/347 | 164/176 | **0.801** |

p, maximum probability obtained in COLONY 2.0 for parental genotype inference averaged across 17 loci.
